# A Literature Review on the Role of Hope in Promoting Positive Youth Development across Non-WEIRD Contexts

**DOI:** 10.3390/children10020346

**Published:** 2023-02-10

**Authors:** Rachael M. Bowers, Edmond P. Bowers

**Affiliations:** 1 Institute for the Advancement of Community Health, Furman University, Greenville, SC 29613, USA; 2 Youth Development Leadership, Clemson University, Clemson, SC 29634, USA

**Keywords:** adolescence, agency, cross-cultural, hope, pathways, positive youth development

## Abstract

Theoretical and empirical evidence has identified hope as a key adolescent strength that is linked to positive youth developmental outcomes. Although hope must be understood from a cultural lens, most of the evidence regarding adolescent hope is derived from samples of white youth from Western, educated, industrialized, rich, and democratic (WEIRD) nations. Therefore, in order to have a more comprehensive and global understanding of the antecedents, outcomes, and processes of hope, we apply a positive youth development perspective in order to review the literature (N = 52 studies) on hope from diverse cultural and international contexts. In organizing the findings by global region, our review provides evidence of the shared function of hope in promoting positive youth developmental outcomes and the usefulness of the Child Hope Scale across contexts. Family and parental relationships were identified as key assets in promoting hope; however, there exists cultural and contextual variation in what aspects of these relationships promote hope. We conclude this review by considering the priorities for research, practice, and policy, based on these findings.

## 1. Introduction

Hope has been the subject of study within multiple disciplines, including nursing, philosophy, psychiatry, theology, and psychology [1]. Erikson [2] recognized hope as a product of the first of his stages of psychosocial development, and Snyder and colleagues [3,4] can be credited with developing the most robust theory on hope and in spurring the empirical study of hope in the field of psychology. Other psychologists have worked on the construct of hope throughout the latter half of the twentieth century and have developed a variety of conceptual definitions, including hope as a condition response, as a goal expectation, and as a spiritual attachment [1]. In contemporary research, hope has been examined through the frameworks that are provided by relational developmental system (RDS) theory [5] and, for adolescent hope, from the positive youth development (PYD) perspective [6,7].

The conceptual emphasis in RDS-based theories is placed on mutually influential relationships between individuals and contexts (i.e., person ←→ context relations). These relations regulate the course of development. When these “developmental regulations” involve person ←→ context relations benefitting both the person and his or her ecology, they may be termed as “adaptive” [8]. 

Derived from RDS metatheory, the PYD perspective is a strength-based approach to adolescence that posits that all youth have individual strengths and the contexts within which youth live possess resources for their healthy development. There are several models that are associated with RDS-based ideas that have been used to study PYD [9]. As the models of PYD are founded on the RDS conception of adaptive person ←→ context relations, they posit that, when the strengths of youth are aligned with the resources that are found in the key contexts of youths’ lives, youth are more likely to thrive. From the PYD perspective, hope is a key youth strength. When hope is aligned with the resources that are present in a young person’s context (e.g., families, schools, or afterschool programs), mutually beneficial bidirectional person ←→ context interactions result, which in turn, lead to healthy, thriving, and contributing young people [6]. The strength-based emphasis on youth as “resources to be developed” counters the deficit-oriented approach that has defined much of the history of research and work with adolescents [9]. 

With an emphasis on mutually beneficial person ←→ context relations, the PYD perspective stresses person-centered approaches. That is, no two youth share the same person ←→ context relations across their lives, and, therefore, each youth’s life trajectory will be specific to that unique individual [10]. The focus of scholarship and practice must be on the individual [11]. Thus, in order to understand positive and healthy development in young people, one must understand the role of social and cultural contexts in that development [12,13]. Indeed, PYD is culturally defined ([12,14], and family, culture, and society shape youth strengths, their positive future expectations, their goals, and the potential barriers to those goals [15,16]. However, despite the growing focus of many youth researchers and practitioners on the role of hope in promoting PYD, much of this work has emerged from studying white youth in the context of Western, educated, industrialized, rich, and democratic (WEIRD) nations [6,17]. The PYD perspective has become globalized in recent years [12,18], but very little is known about the links between hope and thriving in adolescents from diverse cultural, ethnic, religious, and socioeconomic backgrounds [6,15,19]. How hope is defined and conceptualized may differ across cultures and contexts, and the potential antecedents and outcomes may also differ [6]; that is “all aspects of hope exist in a cultural context” [15] (p. 99). Therefore, we review the empirical work on hope and positive development in adolescents that has been framed by Snyder’s hope theory [20] and based on data that are derived from samples of non-white youth in the US and from Europe, Africa, the Middle East, Oceania, and Asia. The goals of this review are to examine how hope functions within and across contexts in order to identify contextually specific versus universal processes, to identify gaps in the study of hope from a cross-cultural perspective, and to provide implications for research, practice, and policy, based on the existing evidence.

### 1.1. Snyder’s Hope Theory

Snyder [3,20] has provided the most frequently used theory and measures of hope and is credited with developing hope theory. Snyder’s hope theory conceptualizes hope as consisting of two components, which are pathways and agency. Pathways are a set of beliefs in one’s abilities to generate one or more paths to desired goals and are generated so that individuals can avoid or overcome obstacles. Agency involves the perceived motivation to attain said goals [3,4,20]. Hope is closely related to other positive psychological constructs, including optimism, self-efficacy, and problem solving. Those constructs give different emphasis to the goal itself (i.e., optimism and self-efficacy), to future-oriented agency, or to pathways-related processes (i.e., problem-solving), whereas Snyder’s hope theory equally emphasizes each of these goal-pursuit components [21]. According to hope theory, a goal can be anything that an individual desires to experience, create, obtain, carry out, or become, and hope requires the presence of both personal agency and pathways from the individual for the successful pursuit of the goal [22].

Snyder and colleagues have also developed measures that aligned with their conceptualization of hope. These measures are the Adult Dispositional Hope Scale, the Adult State Hope Scale, and the Children’s Hope Scale [22]. The Adult Dispositional Hope Scale and the Adult State Hope Scale differ in the sense of the time that is used as a prompt for the participant. The former asks the respondents to describe themselves when completing the items and the latter asks the respondents to describe themselves at a particular moment in time (Snyder, 2000). Both of these are brief, including 12 items, and both can be used with adolescents from the age of 16 years and older and throughout adulthood [22]. The Children’s Hope Scale can be used with children who are as young as 7 years to 14 years and is composed of six items [22].

Although the components of hope theory may be universal, the desired goals that the youth set, the pathways that they pursue, and the obstacles that they face in these pursuits can be influenced by culture and context [15]. Hope is defined and operates in different ways across different people [23]. Expansions and modifications of Snyder’s theory have emerged from different cultural contexts beyond the predominately white/Caucasian American context of Snyder’s work; however, whereas Snyder [20] highlighted the rainbow as a symbol of hope, much work is still needed in order to define the colors of that rainbow for diverse adolescents [17]. 

### 1.2. Hope in Adolescence

Higher levels of hope are consistently related to better outcomes in a variety of areas of life [20]. Hope is particularly important during adolescence, as it is during this stage of development that a sense of a personal future comes to the fore and identity formation becomes an important task [24]. Many physical, psychological, emotional, and cognitive changes define adolescence. As adolescents go through the physical changes that are brought on by puberty, their social contexts also change. Youth develop new and diverse relationships beyond the family, with peers becoming much more important [25]. Neurobiological development occurs along with the associated growth in cognitive abilities that are marked by formal operational thought, abstract thinking, and hypothetical deductive reasoning [26]. As youth become more attuned to their environment, they also seek autonomy and independence [27], progressing from childhood to adulthood, as society redefines their position. The co-occurrence of individual and contextual changes makes thriving complicated. Therefore, a key task during this period is to organize these changes into a definition of the self that will help to prepare the individual for the future [7]. Hope can serve as a psychological strength that motivates youth to achieve this task and look toward the future. Within PYD theory and research, hope has been identified as an individual strength that influences PYD and the development of a positive path to adulthood [7]. For example, higher levels of hope are significantly linked to higher PYD, contribution, and lower levels of risk behaviors and depressive symptoms in adolescence [28]. 

Hope has also been specifically shown to have a positive relationship with academic success in high school [29] and psychological well-being, in terms of personal adjustment and life satisfaction [30]. Evidence has demonstrated that hope can also be a protective factor for adolescents who are at risk. Studies have shown that hope has moderated the impact of the exposure to violence [31] and stressful life events [32] and has been associated with a reduced risk for suicidal ideation for adolescents [33]. Although the evidence of the relationship between hope and positive adolescent development is strong, the overwhelming majority of the evidence regarding hope is from samples of youth from WEIRD contexts [15,17]. As PYD approaches become more common across the globe, there is a great risk that the findings and the perspectives from WEIRD contexts may dominate. Thus, there is much room for a deeper understanding of how hope functions in relation to an adolescent’s culture and context across youth from non-WEIRD contexts. 

In recognizing the role of culture and context in the development of hope, and the functionality of hope in promoting healthy and positive development, it is important to consider the specificity principle [11,34]. The specificity principle indicates that developmental phenomena are best understood in light of the specific characteristics of the individuals in relation to the specific characteristics of the contexts in which they are embedded [10]. In applying the specificity principle, one can identify the commonalities across people and contexts, but also better understand the heterogeneity that exists across individuals. A better understanding of both the specific and the common relationships between hope and PYD outcomes in non-WEIRD contexts may help scholars and practitioners to identify both universal and contextually relevant ways to promote thriving in youth globally. 

Therefore, the focus of this paper is a review of the literature on hope in varying non-WEIRD cultures/contexts. We aim to synthesize the results of these studies in order to identify the following points:

1. Where the findings converge (i.e., universality/commonality);
2. Where the findings diverge (i.e., cultural/contextual specificity);
3. Where gaps remain in the literature.

As Chang and Banks [17] reflected, Snyder [20] highlighted the iconic use of the rainbow to symbolize hope, however, much work is needed in order to define the colors of that rainbow.

## 2. Methods

The present review includes peer-reviewed articles that were available online between the years 2000 and 2021, in order to align with the period when the PYD perspective became prominent in the study of adolescence [35,36]. We do not present the research reviewed in this section as an exhaustive list, as our goal was not to provide an extensive review, but instead to highlight evidence in support of hope as a strength for youth from diverse non-WEIRD contexts and to suggest the possible implications for research, practice, and policy, based on this evidence. The initial search for the literature for this review was conducted through Google Scholar and PsychInfo. Using the search terms “hope theory” “adolescents” and “cross cultural”, 454 results were generated. To complement this initial search, we also relied on our familiarity with the work of PYD and hope scholars; therefore, our search also included these readings. In addition to reviewing these bodies of evidence, we also conducted forward and backward searches in order to find additional articles. For the backward search, we carefully examined the reference sections of the initial articles. For the forward search, we reviewed studies that cited the papers in our initial body of evidence using the ‘cited by’ feature in Google Scholar and PsychInfo.

The articles included in this review met the following criteria:

1. The articles had a publication date between 2000 and 2021, or were published online as of 11 November 2021;
2. Available in English;
3. Adolescents were the primary study sample, and the mean sample age was between 10 and 18 years of age;
4. Hope was a dependent or independent variable of study;
5. The sample population did not encompass a majority white U.S. sample;
6. The studies were framed by Snyder’s hope theory [20].

We selected 18 years of age as the upper age limit for the study inclusion criteria due to adolescence being defined as the period from 10 to 18 years [37], the heavy presence in the hope literature on studies of adult and collegiate samples [38], the importance of hope in adolescent development [6], and the emphasis on the second decade of life in studies framed by the PYD perspective [13]. We focused only on studies that were framed by Snyder’s hope theory, as this theory is the most used and well-supported model for conceptualizing and studying hope [15]. Hope theory, therefore, provides a frame for comparing and contrasting across cultures and contexts in order to identify contextually/culturally specific and universal mechanisms of hope. This review process led to the identification of 52 studies for our review (Table 1).

## 3. Results

The identified studies were grouped largely according to the designated United Nations’ geographic regions of the world, as follows: United States minority groups (i.e., Northern America), Europe, Africa, Oceania, the Middle East, and Asia. No studies from Latin America or the Caribbean met the inclusion criteria. Table 1 provides the region and country, the sample age, and the role of hope for which the qualifying literature was reviewed. Studies from Asia, particularly those from China, and the United States reflected the greatest proportion of studies in our review. A relatively equal number of studies examined hope as a predictor (16 studies) or as an outcome (19 studies) of the PYD processes. Fewer studies considered the role of hope in a more complex manner, as only four studies examined hope as a mediator of developmental mechanisms and only five considered how hope might moderate phenomena; in addition, only four studies took a qualitative approach in order to explore hope processes more fully. Finally, eight studies aimed to validate Snyder’s Child Hope Scale in diverse populations.

### 3.1. Hope among Minority Adolescents in the United States

Although research in minority adolescents in the United States has linked hope to positive outcomes, particularly for African American and Latino youth, hope remains understudied for these groups and other ethnic minorities [15]. Research to date provides three summative points in support of Snyder and colleagues’ work. First, Snyder et al.’s [3] measurement tools have validity with minority adolescent populations in the United States [42,45]; second, the agency and pathways constructs of Snyder’s theory correspond to the understanding of hope for these ethnic groups [43]; and third, hope functions to benefit minority youth in the U.S. in similar ways as in majority white youth, particularly in terms of academic success [39,41,44].

Research with minority youth in the U.S. points to the protective effects of hope in the face of multifaceted inequities and obstacles. For example, Roesch and colleagues [45] conducted a comparative study of hope with a predominately Latino sample that also included Asian American, African American, bi-racial, and Native American adolescents. They found that high levels of hope were associated with the use of a greater number of overall coping strategies to manage daily stressors in these youth. The pathways component of hope was specifically found to be associated with problem solving, planning, positive thinking, and religious coping. The authors concluded that hope served as a protective factor against the negative impact of the daily stress that is faced by minority adolescents.

In samples of youth that are often marked by acute risk and stress, Twyford, Dowdly, and Sharkey [47] determined that Latino adolescents who were on probation, irrespective of their gender, reported lower hope than non-probation comparative samples, and that hope showed a significant correlation with a reduced risk for recidivism. Hope was also a protective factor for African American fifth graders who had been exposed to, or were victims of, violence, as hope moderated the effect of the violence on their self-concept and personal adjustment [31].

Several studies have also pointed to the role of hope in academic success and achievement in minority youth in the US. For example, in McCoy and Bowen’s [44] study of predominately African American and Latino adolescents in Chicago, the authors found that higher levels of hope were related to increased self-efficacy in school. Adelabu [39] also identified the benefits of hope for academic success in rural African American adolescents, whereas Vela, Lenz, Sparrow, and Gonzalez [48] found that hope predicted college attendance beliefs in Mexican American adolescents. Dixon and colleagues [41] examined hope as a mediator for the relationship between SES and grade point average in a general sample of youth and a targeted sample of ethnic minority youth. In both of these populations, the results indicated that hope partially mediated the relation, suggesting that hope had a consistent influence on grades that was not affected by the additional strain and stress that is experienced by minority youth. 

Research on minority youth in the US has also pointed to several antecedents that are promotive of hope. Harley [43] conducted a qualitative study of the factors that contribute to the development of hope for 16 African American adolescents living in a low-income Midwestern neighborhood using both photovoice and semi-structured interviews. The analysis identified five themes that were related to sources of hope—caring connections, education, spirituality, “the basics” and a “gonna-make-it mentality”. Harley used “the basics” in order to capture the participants’ sentiment that having one’s basic needs met (i.e., food, shelter, and clothing) was a source of hope. Additionally, a “gonna-make-it mentality” referred to the mental toughness and the perseverance that were expressed by the participants, which is similar to Snyder et al.’s concept of agency [3]. Studies of hope in minority adolescents have also found that neighborhood safety and supportive parental relationships contribute to the maintenance of hope for urban minority youth [44], and there was a positive correlation between maternal support and hope in African American females [40]. Edwards, Ong, and Lopez [42] found that hope scores positively correlated with support from family and friends, as well as positive affect, life satisfaction, and optimism in Mexican American adolescents. 

Shadlow, Boles, Roberts, and Winston [46] published the sole study that is available regarding hope and Native American adolescents. This study determined that Native American adolescents also conceptualize hope as a way to reach their goals. However, agency was shown to be a more stable construct than pathways for this population, as only two of three items measuring pathways loaded accordingly in the factor analysis. The authors suggest that the Native American children in the sample may feel less able to reach a goal due to their own or their family’s history of experiences with discrimination.

### 3.2. Hope among European Adolescents

The research on hope in European contexts is limited and scattered across the continent. Of the eight studies that have been reviewed here, four derived data from Portugal, two from Italy, one from Croatia and Serbia respectively, and one compared Italian and Swiss samples. The influences of economic, educational, and religious contexts are highlighted in the research from Europe regarding hope and adolescents. Although it is limited, the evidence from the European contexts does demonstrate the importance of considering individual and contextual factors in relation to hope. For example, in a study of career adaptability in the context of poor economic circumstances following the global financial crisis of 2009, Santilli, Marcionetti, Rochat, Rossier, and Nota [56] compared samples of Italian and Swiss adolescents. They found that hope partially mediated the relationship between career adaptability and life satisfaction in the Italian adolescents and fully mediated the relation in the Swiss adolescents. These results point to the critical role that economic and educational contexts play on the relationships between these variables. In addition, Falanga and colleagues [50] provide support for the individual factors that are linked to hope, as they found that adolescent use of positive humor styles that promote social relationships and personal well-being were positively related to both the agency and the pathways dimensions of hope. 

Work with Portuguese adolescents has identified relationships between hope and life satisfaction and hope and academic achievement that are consistent with the findings from other cultural contexts. In work with high school students, Marques, Lopez, and Mitchell [52] found that hope and spirituality were predictive of life satisfaction one year later. It is important to note that the Portuguese rank as highly religious and highly satisfied with life as compared to other European peoples [88]. Marques, Pais-Ribeiro, and Lopez [53] also found that hope predicts academic achievement two years later in a sample of middle school Portuguese students. Marques, Lopez, and Pais-Ribeiro [51] also developed a five-week intervention in order to help youth to identify clear goals and pathways to attain those goals, as well as to promote youth motivation to persist and skills to reframe the obstacles to those goals. The results indicated that the intervention enhanced short- and long-term (i.e., 18 months) youth hope, life satisfaction, and self-worth. The positive influence of hope on adolescent outcomes also extends to specialized populations of the Portuguese youth. Martins et al. [54] found that hope had direct and indirect effects on the health-related quality of life for youth with a diagnosis of malignant cancer. Their results suggest that hope is a psychological strength that is linked to the quality of life directly and via its effect on youth anxiety. 

Tests of the validity for the Children’s Hope Scale have been conducted with youth in Serbia [38], Croatia [55], and Italy [49]. In a sample of Serbian adolescents, Jovanović [38] established convergence validity with the agency dimension more closely related to optimism and self-esteem and with the pathways dimension more closely related to self-efficacy and resilience. In the results that were derived from a sample of Croatian adolescents, Merkaš and Brajša-Žganec [55] indicated a unidimensional structure of hope. After constructing clusters of low-hope and high-hope children, they determined that high-hope children reported greater life satisfaction, self-esteem, social support, and family cohesion. Alfieri and colleagues [49] found excellent fit for Snyder’s two-factor model of hope in a sample of Italian adolescents, which was invariant across gender and showed convergent validity with a measure of dispositional optimism. 

### 3.3. Hope among African Adolescents

The sparse body of literature from Africa presents a picture of hope among African adolescents that is consistent with the evidence that has been found in other parts of the world. Synthesizing the findings from both qualitative and quantitative research provides support for the applicability of Snyder’s theory, the protective nature of hope, and the links between spirituality and hope. For example, using a qualitative approach in order to understand the sources of hope for Ghanaian adolescents, Wilson and Somhlaba [62] conducted 18 interviews with adolescents who were living in low socioeconomic neighborhoods in urban and semi-urban Northern Ghana. Despite the poor living conditions, the authors found that the adolescents in this sample expressed high levels of hope that were associated with their religious tradition, their family relationships, and their relationships with their teachers and peers. 

The relationship between hope and residential stability was explored in Nalkur’s [60] mixed-method study of adolescent boys from three youth subcultures in Tanzania—street youth, former street youth, and school youth. This study found that the stability of youth living contexts influenced their perceptions of hope. The youth in unstable environments (i.e., those living on the streets or homeless) tended to avoid hope in order to protect themselves from failure, and they were inclined to attribute success to luck or external factors. The youth who formerly lived on the streets reported that, after they had shelter and felt safety and a sense of belonging, they were able to develop supportive relationships and a belief in God in order to bring about hope and pursue their goals. The youth in stable environments (i.e., in the presence of supportive adults, living at home, and attending school) focused on internal resources and saw themselves as the producers of their own hope. 

Work with a similar sample of at-risk youth in South Africa is suggestive of the importance of hope in the contexts of violence and poverty [59]. In focus groups with 14 adolescents from Cape Town, the authors found that the participants conceptualized hope as a future-oriented, protective factor with high importance, due to its link to their faith and for its value for coping with the challenges of violence and poverty in the community [59]. Other studies of South African adolescents have validated the Children’s Hope Scale [58], have found that hope is linked to psychological well-being [57], and have identified hope as a stronger predictor of well-being than community violence [61].

### 3.4. Hope among Adolescents in Oceania

Some of the most substantial longitudinal work on hope in adolescents comes from scholars in the Oceanic region of the world. This work offers critical evidence of the need to support hope in adolescents so that hope and the associated positive outcomes may be maintained throughout adolescence. Much of this work has come from the *Wollongong Youth Study*, which is a longitudinal study that was conducted with students who were enrolled in Catholic schools in grades 7 through 12 in the Diocese of New South Wales, Australia. Using the first two waves of data, Ciarrochi, Heaven, and Davies [63] found that hope was a better predictor of grades a year and a half later than self-esteem or positive attributional style. In subsequent work, Heaven and Ciarrochi [65] demonstrated a general decline in both hope and self-esteem over grades 7 to 10. Although females initially reported higher hope, they exhibited a more rapid decline than males. Reported baseline parental authoritativeness was related to higher levels of hope across all four years for both genders.

Ciarrochi, Parker, Kashdan, Heaven, and Barkus [64] also examined the relationship between hope and its affect in grades 7 through 12. The results showed that hope predicted positive affect, but positive affect did not predict hope. Hope and negative affect were reciprocally related, as hope predicted lower sadness, fear, and hostility, while sadness, fear, and hostility predicted lower hope. Finally, hope made the largest contribution to future well-being during the school transition years. In affirming the need to support youth hope over adolescence, Phan [66] also studied the development of hope and academic self-efficacy in a sample of Year nine students from the Fiji Islands. Over a two-year period, their hope increased, flattened, and subsequently decreased. Hope and self-efficacy were also complexly related. The youth who reported higher initial self-efficacy beliefs for academic learning were also more likely to experience a more rapid increase in hope; however, the initial levels of self-efficacy were negatively related to the initial levels of hope.

### 3.5. Hope among Adolescents in the Middle East

The research findings on hope from the cultural contexts in the Middle East vary somewhat from the findings in the United States, Europe, and Africa. Most notably, the evidence suggests the impact of violent contexts on the levels of hope differs and suggests that hope may not be a universally protective factor in the presence of persistent violence. Sagy and Adwan’s [73] study of Israeli and Palestinian adolescents provides an important foundation to begin the consideration of hope in a Middle Eastern context. They suggest that hope should also be understood with consideration of the impact of contextual (i.e., persistent violence) and cultural (i.e., individualistic versus collectivist orientations) influences [73]. With a sample of tenth and twelfth grade students from Israeli and Palestinian communities, the authors examined individual hope (hope for self) and collective hope (hope for others) in the context of national conflict. Although the Palestinian sample scored higher on hope for others than the Israeli sample, both of these groups prioritized hope for self over hope for others. The authors noted that both of the groups shared mid to high socioeconomic status, which may have contributed to the higher personal hope scores. Furthermore, the authors observed that, although the levels of hope were high, the adolescents in this sample reported a low value of peace; they suggest that growing up in a context of historic and persistent conflict contributes to a low potential for a vision of a peaceful future. In order to further support the impact of persistent violence on hope, Braun-Lewensohn and Sagy [67] surveyed Israeli adolescents using hope as a proxy measure of adolescent spirituality to examine the threat and the presence of missile strikes on spirituality. The authors found that hope, a sense of coherence, and collective values decreased in the context of a prolonged exposure to violence.

Haj-Yahia and colleagues examined the links between personal experiences of violence, that is, interparental conflict and maltreatment, on youth psychosocial outcomes among Arab adolescents living in Israel [89,90]. The youth who witnessed violence and aggression between their parents, or those who were physically and psychologically maltreated, reported higher levels of hopelessness. Additionally, in the context of Iranian family life, Khodarahimi [71] found that the experience of family violence had a negative correlation with both hope and mental health for adolescents. In contrast, in a study with 300 adolescents in Iran, Sadeghi and colleagues [72] found that the levels of differentiation of self can predict hope, as mediated by resilience. That is, the adolescents who strike a healthy balance of autonomy and connectedness to other people are more likely to bounce back from adversity, which in turn, promotes higher levels of hope.

Several findings from the Middle East were derived from samples of Turkish youth [68,69,70,74]. For example, Karababa [69] found that hope was negatively related to both maladaptive perfectionism and anxiety in a sample of Turkish early adolescents. In addition, hope served as a protective factor for the relationship between maladaptive perfectionism and anxiety; that is, when youth reported lower hope, the relationship between maladaptive perfectionism and anxiety was stronger.

The studies with Turkish youth also considered the links between parenting and hope. Sahin-Baltaci [74] compared Turkish and American youth on levels of hope and life satisfaction. Sahin-Baltaci found that the levels of hope in the Turkish adolescents did not differ from their American counterparts; however, the Turkish students reported higher levels of life satisfaction. Sahin-Baltaci [74] also found that, whereas hope in the Turkish youth was positively related to youth SES, but was not related to parenting attitudes, the opposite relations held true for the American youth. The American youth did not differ in hope by SES level, but those youth who reported democratic parenting attitudes were more likely to report higher hope and life satisfaction than the youth who reported authoritarian or overprotective parenting. The authors suggest that these cultural differences may be related to how the meaning of authoritarian and overprotective may be viewed positively in Turkish families. Kemer and Atik [70] examined the relationship between the perceived family social support and hope in rural and urban high school students in Turkey. The authors concluded that the love- and esteem-related components of family social support were related to the highest levels of hope, and that hope was higher for males compared to females. Çelik, Çetin, and Tutkun [68] examined the relationships between personal and familial resilience factors and hope in a large sample of male soccer players from low socioeconomic communities in 10 Turkish cities. The results indicated that personal (self-efficacy and self-awareness) and familial (support and communication) factors were associated with elevated hope, while school and peer contexts were not. The authors suggest that the lack of a significant relationship between hope and school and peer contexts may be due to the strict and rigid nature of Turkish educational institutions.

### 3.6. Hope among Asian Adolescents

The research findings on hope in adolescents in Asia have been consistent with those from other parts of the world regarding the protective and adaptive functions of hope. This work has primarily been based on samples of youth from China, as the studies from China were the second most reviewed after those that were derived from US samples. For example, using a large sample of 10th and 11th grade students in China, Ling and colleagues [81] demonstrated that hope was positively related to self-esteem and optimism. Additionally, this study found that self-esteem and optimism both partially mediated the relationship between social support and hope. Ling and colleagues [21] also looked at the relationship between hope and the outcomes of life satisfaction, self-esteem, optimism, and depression in a sample of Chinese youth in grades 8 to 11. After clustering the respondents into “high hope”, “average hope”, and “low hope” groups, the authors determined that the “high hope” group reported significantly higher levels of life satisfaction, self-esteem, and optimism, and significantly lower levels of depression.

Three studies have been conducted in order to extend the understanding of how hope functions in adolescents in Hong Kong [77,80,87]. Yeung, Ho, and Mak [87] found a significant positive relationship between hope and happiness and a significant negative relationship between hope and anxiety, depressive symptoms, and interpersonal difficulties. Supporting the links between hope and self-regulation [6], these relations were partially mediated by attention to positive information. Chai, Kwok, and Gu [77] and Kwok and Gu [80] considered the role of hope as a protective factor against maladaptive outcomes in the youth from Hong Kong. Chai et al. [77] considered both psychological (hope and life satisfaction) and contextual (autonomy-granting parenting) resources in predicting youth depression. As expected, these resources negatively predicted depression. In addition, hope and life satisfaction moderated the relationship between parenting and depression. That is, higher youth hope and life satisfaction protected youth against higher levels of reported depressive symptoms under lower levels of autonomy-granting parenting. In a similar way, Kwok and Gu [80] found that hope was negatively related to suicidal ideation, and that hope buffered the indirect effect of childhood neglect on suicidal ideation via depressive symptoms. 

Similar benefits of hope were also found in other Asian countries. Wong and Lim [84] showed that hope and optimism were positively correlated to each other in a sample of adolescents in Singapore; both of these constructs also predicted lower levels of depression and increased reports of life satisfaction. Regression analyses showed that optimism and pessimism were predictors of depression, but the pathways component of hope was not. The authors have suggested that the greater government control of one’s pathways in Singapore may have contributed to a collectivist rating of hope rather than an individual one. Botor [76] also found that hope benefited Filipino adolescents who were participating in an organized youth program. Both the agency and the pathways dimensions of hope predicted resilience, but only agency was a positive predictor of general happiness and overall personal well-being. 

Several studies from Asia have also explored how interpersonal relationships are linked to adolescent hope, with mixed results [79,83,85]. Xiang and colleagues [85] examined how self-concept clarity and hope mediated the relationship between family cohesion and subjective well-being in a sample of 2792 Chinese adolescents and early adults. They found that both of these psychological resources served as independent mediators of the relationship, and that the extent to which the family members were concerned and committed to the family could influence youth well-being through the chain mediating effect of “self-concept clarity–hope” [85] (p. 81). Izzaty and Ayriza [79] identified similar benefits of family resources in their exploration of the link between parental bonding and hope in 400 Indonesian adolescents. They found that warm and loving care and autonomy-granting from each parent were predictive of adolescent hope, whereas overprotective parenting was not. Beyond the family context, Nie et al. [83] found that supportive teacher–student relationships were positively linked to life satisfaction and hope in a longitudinal study of 1108 adolescents from southwest China. Hope was also found to mediate the association between teacher–student relationships and life satisfaction, both between people at one point in time and within a person longitudinally. That is, more positive teacher–student relationships predicted hope, which in turn, predicted life satisfaction.

In contrast to these findings, Chui and Wong [78] studied the relationship between hopelessness, happiness, and life satisfaction with students in the context of the increasing divorce rates in Hong Kong, with surprising results. For the adolescents whose parents were separated, the authors reported decreased life satisfaction and happiness, but a stronger sense of purpose in life, a higher self-esteem, and lower hopelessness compared to the youth whose parents remained married. The authors suggest that adverse circumstances, such as those that are associated with divorce, force the adolescents in Hong Kong to focus more on becoming independent and forming their own.

Efforts from Asia have also supported the use of the Child Hope Scale with Asian adolescents. Yang et al. [86] assessed the measurement invariance of the Children’s Hope Scale in samples of Chinese and American adolescents. They found that, whereas the two-factor model that was proposed by Snyder provided a better fit for the American adolescents, a one-factor model exhibited a better fit for the Chinese sample. Yang and colleagues suggest that these findings may be due to the cultural differences in cognitive styles, as Chinese youth conceptualize hope as a whole, whereas American youth distinguish between the agency and the pathways dimensions, due to the individualistic values that are emphasized in personal goals. The authors also caution that researchers should not make mean-level comparisons between the two factors of agency and pathways in Chinese and American adolescents. Conversely, Nazam and Husain [82] assessed the validity of the Child Hope Scale for use with Indian youth, with the findings largely supporting Snyder’s original two-factor structure of the scale, except for two of the items that did not load on their original factors. In a study of adolescents in the Philippines, Bernardo [75] extended Snyder’s theory to include internal and external locus-of-hope constructs. Internal locus-of-hope identifies the individual as the agent of goal-attainment cognitions, whereas external locus-of-hope suggests external forces as the agent of goal-attainment cognitions. Moreover, external locus-of-hope further differentiates into three sub-dimensions—family, peers, and spiritual beings or forces (e.g., God, Allah, other deities, or fate).

## 4. Synthesis of Findings

The specificity principle [11,34] points to the specific characteristics of individuals and contexts that co-act in order to define the processes of human development. Approaching the study of hope and PYD with the specificity principle in mind can help us to identify the phenomena that are common across individuals and contexts and those that are unique to individuals and/or contexts. Considering the findings across these global regions, the following three key commonalities regarding hope in adolescence can be ascertained: (1) hope has consistent benefits for diverse PYD outcomes; (2) familial relationships are essential for hope; and (3) Snyder’s Child Hope Scale is a reliable and valid measure of hope in adolescence. Across geographical regions, hope was shown to be predictive of diverse indicators of PYD, including psychological well-being, life satisfaction, health-related quality of life, positive affect, self-efficacy, self-esteem, optimism, problem solving, coping with stress, happiness, interpersonal skills, and academic success in non-WEIRD contexts. In addition, hope served as a protective factor that buffered “risk-immersed” youth from maladaptive outcomes, including youth on probation [47] or those that have been exposed to violence [31] in the US; youth living in communities of poverty and violence in Africa [59]; and youth who have experienced childhood neglect in Asia [80]. Youth experiencing high levels of need were only able to experience hope after their basic needs, such as food, shelter, and safety, were met [43,44,60].

The explorations of the antecedents of hope indicated that caring connections and, in particular, supportive family relationships, were shown to be a consistent predictor of hope in the United States [40,42,44], Ghana [62], Australia [65], Turkey [68,70], China [85], and Indonesia [79]. These findings are consistent with Callina and colleagues’ model of hope highlighting connectedness [6], as well as Snyder’s contention that hope is acquired from the environment that is provided by the important people in their lives [91].

This review also provided evidence for the application of Snyder’s Child Hope Scale [3] as a psychometrically sound measure of hope across diverse global regions, including Northern America [42,45,46], Europe [38,49,55], Africa [58], and Asia [82,86]. Our review, however, found no evidence of the validation of the Child Hope Scale in Oceania, the Middle East, Latin America, or the Caribbean.

In addition to Snyder’s hope theory helping us to identify the universal processes across non-WEIRD populations of youth, we were also able to identify specificities due to cultural and contextual differences, particularly around the influence of the family context on hope [74]. For example, the context moderated whether hope served as a protective factor for the exposure to family conflict. Family violence was positively linked to hopelessness in Middle Eastern adolescents [69,89]; however, hope remained protective despite the experience of parental separation for adolescents in Hong Kong [78] and neglect in China [80]. Perhaps the protective impact of hope is not enough when youth are exposed to stressors at both the individual and the collective level. Indeed, experiencing PYD may be particularly challenging in the face of abrupt, major, or traumatic nonnormative events, such as the political and community violence that is experienced by youth in the Middle East [92]. 

This review has also revealed support for the conceptualization of hope as a spiritual attachment [1], as several studies have pointed to the important connection between hope and spirituality in the lives of youth, especially those facing adverse and unstable life situations, such as youth living in some communities of the Middle East [67]. Spirituality has been identified as a contextually relevant strength for youth of color in the US, as these youth tend to be particularly religious [93], which is consistent with Harley [43], pointing to its significance for hope in African American youth living in low-income communities. Youth in Northern Ghana have also described how religious traditions are promotive of their hope [62], and hope in Filipino youth was identified to include spiritual forces as a key source of support for their goal attainment [75].

In addition, the dimensions of hope—agency and pathways—functioned differently across contexts. In most contexts, both of these dimensions of hope predicted positive outcomes in adolescents, however, that was not the case in several Asian countries. Wong and Lim [87] and Botor [76] found that the pathways component of hope was not related to adaptive outcomes in the youth in Singapore and the Philippines, respectively. In addition, Yang et al. [86] found that the agency and pathways components did not differentiate in Chinese youth, as was found in the other Child Hope Scale validation studies. The authors of these studies suggested that culture influenced these unanticipated findings, as the emphasis on collectivist orientations may limit the role of pathways in the lives of the youth in Singapore, the Philippines, and China.

Looking across the 52 studies has also revealed several gaps in the literature on hope in adolescents from non-WEIRD contexts. Circling back to Chang and Banks’ [17] rainbow metaphor, some of the colors of the rainbow have been filled in regarding the understanding of hope in global contexts. There remains gray, however, regarding what promotes hope outside of family contexts; the interrelations between hope and spirituality; and hope within Latin America and the Caribbean. Moreover, the study of hope requires additional attention in many parts of the world.

Although the evidence points to the family as a key setting for promoting hope and PYD, additional contexts must be considered. Only a few studies have explored relationships beyond the family context [83]. Nie and colleagues [83] identified teacher–student relationships as being promotive of hope in adolescents, and youth development programs have been identified as contexts that support hope [6]; however, little research has examined these contexts within or across cultures and contexts. As these programs begin to serve more diverse youth and families [94], incorporating culture and context into the implementation of these youth-serving programs can be integral to their success overall and in promoting hope in youth [12,94].

This review has also illuminated the substantial connection between hope and spirituality for adolescents, especially within contexts of violence [43,59,60,62]. As indicated, the Middle East stands out as a region where a joint context of violence, both at the national and the family level, has been linked to diminished hope. As hope and spirituality were connected in communities of color that were under the threat of violence in the United States and Africa, the role that religion plays in persistent national conflicts in these regions may be confounding the function of hope for adolescents.

Geographically speaking, a great deal of work remains to be carried out in understanding hope. No literature was found from the context of a Latin American, Caribbean, or South American country, and the sparse number of articles that were found from Europe, Africa, Asia, and Australia do not reflect the diversity of the cultures and contexts within each region. In addition, only a few of these studies considered cross-cultural approaches in their designs [56,74], limiting our ability to provide suggestions and implications without caution. The growth of the study of hope in a greater number of cultural contexts would help us to better understand how hope exists as a universal or context-dependent phenomenon.

## 5. Implications for Research, Practice, and Policy

In terms of the implications for future research, we emphasize our point that a great deal of work on adolescent hope is still needed. In addition to relatively few studies being identified in most non-WEIRD contexts, much of the existing work is limited in scope. Most of the studies that were reviewed were cross-sectional in nature and considered hope as a predictor or an outcome in a correlation design. Only a few studies examined hope through longitudinal or qualitative designs that would allow for a greater exploration of the mechanisms by which hope is promoted and promotes PYD outcomes across diverse contexts. Future studies need to employ mixed-method designs in order to understand hope processes more fully.

In addition to the limitations of their designs, most of the studies that have been reviewed here included only one or two outcomes of hope, with the majority focusing on measures of life satisfaction. The studies often only included one contextual antecedent of hope, with almost all of them focusing on the family. A PYD approach to adolescence is based on the relational developmental systems metamodel [5,13], and, therefore, studies of hope must take a more ecological and holistic approach to development. Future studies need to consider multiple potential contextual influences, such as the family, school, and peer group, and more comprehensive indices of PYD, such as the five Cs of PYD [13,14]. The links between collectivist versus individualistic cultural influences and spirituality on adolescent hope [84] are also important to consider from a PYD approach. Therefore, additional research to disentangle the spiritual, individualist, and collectivist perspectives of hope and their impact on youth outcomes across culture and context is warranted. Finally, as indicated, only a few studies included youth from across countries or geographic regions; indeed, few studies even included diverse youth from within a country or region. Therefore, future studies should take a more global perspective and aim to examine the relationships between hope and PYD outcomes across youth from diverse countries and geographic regions.

Although the studies that have been reviewed here have revealed several limitations within the body of evidence on hope in non-WEIRD contexts, our approach to this review is also limited in several ways. The inclusion criteria that were applied to our search limited the ability to explore the full evidence base on youth hope, such as work that was published in non-English publications and in the gray literature published outside of traditional academic outlets. With many scholars, youth-serving organizations, and non-governmental organizations focused on promoting psychological strengths such as hope in youth, future reviews should expand in order to include these important areas of evidence. In addition, we intentionally selected the age range of 10–18 years of age; however, we recognize that this age range does not necessarily align with the definition of youth/adolescence across all cultures and global contexts. Therefore, our findings may reflect how hope operates in youth ages 10–18, but they may not apply to “youth” or “adolescents” in all contexts. Finally, the decision to only include studies that were framed by Snyder’s hope theory excludes publications that include varied definitions of hope or oblique constructs such as “hopeful future expectations.”

Despite these limitations, the findings have several implications for practice and policy. As hope was identified across contexts as a key resource for PYD outcomes, programs and interventions should include activities around identifying goals and pathways to achieve those goals, as well as promoting motivation to pursue those pathways and goals. Across contexts of social change and risk, the promotion of hope may be a key intervention for practitioners and policymakers to focus on when they are uncertain about what actions will have a high probability of promoting resilience or thriving. It is also important that these programs and interventions ensure that a psychologically and physically safe setting is provided, particularly for those programs that are working with youth who have faced adversity or trauma [60]. A consistent body of evidence has also pointed to the important role that family factors play in the promotion of hope. Therefore, programs and interventions should aim to include families in activities and focus on promoting parenting practices that reflect warmth, acceptance, and support of their children. In considering these efforts, practitioners must also be culturally responsive, as the potential goals and pathways may differ across youth cultures and contexts [15,86] and the understanding and acceptability of parenting behaviors also differ across cultures [74,94]

Finally, in turning to the policy implications of the findings, based on the benefits of family social support, teacher relationships, spirituality, and youth development programs for hope, an appropriate community policy would be for the stakeholders in youth development to build systems of support for young people [95]. Multifaceted comprehensive community initiatives [96] may provide the best approach to promote hope in youth who are facing adversity individually and collectively (e.g., maltreated Arab youth in Israel).

## 6. Conclusions

The findings of this review point to the universal benefits of hope for youth living in global regions beyond WEIRD contexts that help us to fill in some of the colors of the hope rainbow [17,20]; however, as opposed to the relatively substantial body of evidence that can be derived from youth in WEIRD contexts, the evidence base for youth living in non-WEIRD contexts is relatively sparse. Additional theoretical, empirical, and practical work in diverse cultures and contexts, both within and across the global regions of the world, is greatly needed in order to contribute to a radiant rainbow of hope.

## Figures and Tables

**Table 1:** Organization of the literature of hope in adolescence by world region.

Author(s)	Country and Sub-Population	Mean Age and/or Age Range (Years)	Role of Hope (Predictor, Outcome, Mediator, Moderator)
U.S. Minority Groups			
Adelabu, 2008 [39]	U.S. African Americans	Mean age = 15; Range: 12–20	Predictor
Cedeno, Elias, Kelly, and Chu, 2010 [31]	U.S. African Americans	Mean age = 10.20	Moderator
Davis-Maye and Perry, 2007 [40]	U.S. African Americans	Mean age = 13.56; Range: 9–19	Outcome
Dixson, Keltner, Worrell, and Mello, 2018 [41]	U.S. Hispanic Americans or Latinos, African Americans, Asian Americans	Mean age = 15.02; Range: 14–17	Mediator
Edwards, Ong, and Lopez, 2007 [42]	U.S. Mexican Americans	Mean age = 14.22; Range: 11–15	Validation of CHS
Harley, 2015 [43]	U.S. African Americans	Mean age = 14.88; Range: 13–17	Outcome *
McCoy and Bowen, 2015 [44]	U.S. African Americans and Latinos	Mean age = 17.2; Range: 15.6–19.9	Outcome
Roesch, Duangado, Vaughn, Aldridge, and Villodas, 2010 [45]	U.S. Latinos, Asian Americans, African Americans, bi-racial, and Native Americans	Mean age = 15.5; Range: 14–18	Predictor
Shadlow, Boles, Roberts, and Winston, 2015 [46]	U.S Native Americans	Mean age = 10.52; Range: 8–14	Validation of CHS
Twyford, Dowdly, and Sharkey, 2014 [47]	U.S. Latinos	Mean age = 15.82	Outcome
Vela, Lenz, Sparrow, and Gonzalez, 2017 [48]	U.S. Latinos	Mean age = 14.47	Predictor
Europe			
Alfieri, Quartiroli, and Baumann, 2021 [49]	Italy	Mean age = 16.07; Range: 12–18	Validation of CHS
Falanga, De Caroli, Sagone, and Indiana, 2020 [50]	Italy	Mean age = 17.60	Outcome
Jovanovic, 2013 [38]	Serbia	Mean age = 16.10; Range: 14–18	Validation of CHS
Marques, Lopez, and Pais-Ribeiro 2011 [51]	Portugal	Mean age = 10.96; Range: 10–12	Outcome
Marques, Lopez, and Mitchell, 2013 [52]	Portugal	Mean age = 16.28; Range: 15–19	Predictor
Marques, Pais-Ribeiro, and Lopez, 2011 [53]	Portugal	Mean age = 11.7	Validation of CHS
Martins, Crespo, Salvador, Santos, Carona, and Canavarro, 2018 [54]	Portugal	Mean age = 13.29; Range: 8–19	Predictor
Merkas and Brajsa-Zganec, 2011 [55]	Croatia	Mean age = 12.7; Range: 10–15	Predictor
Santilli, Marcionetti, Rochat, Rossier, and Nota, 2017 [56]	Italy, Switzerland	Mean age = 14.35	Mediator
Africa			
Guse and Vermaak, 2011 [57]	South Africa	Mean age = 15.1	Predictor
Guse, de Bruin, and Kok, 2015 [58]	South Africa	Mean age = 15.90; Range: 13–16	Validation of CHS
Isaacs and Savahl, 2014 [59]	South Africa	Range: 14–15	Moderator *
Nalkur, 2009 [60]	Tanzania	Mean age = 14.52; Range: 12–18	Outcome *
Savahl, Isaacs, Adams, Carels, and September, 2013 [61]	South Africa	Mean age = 15.04; Range: 13–17	Predictor
Wilson and Somhlaba, 2016 [62]	Ghana	Range: 17–22	Outcome *
Oceania			
Ciarrochi, Heaven, and Davies, 2007 [63]	Australia	Mean age = 12.30	Predictor
Ciarrochi, Parker, Kashdan, Heaven, and Barkus, 2015 [64]	Australia	Mean age = 12.41	Predictor
Heaven and Ciarrochi, 2008 [65]	Australia	Mean age = 12.30	Outcome
Phan, 2013 [66]	Fiji Islands	Mean age = 14.30	Outcome
Middle East			
Braun-Lewensohn and Sagy, 2010 [67]	Israel	Mean age = 15.23; Range: 12–18	Outcome
Celik, Cetin, and Tutkun, 2015 [68]	Turkey	Mean age = 12.32; Range: 9–15	Outcome
Karababa, 2020 [69]	Turkey	Mean age = 12.8; Range: 11–14	Moderator
Kemer and Atik, 2012 [70]	Turkey	Range: 14–19	Outcome
Khodarahimi, 2014 [71]	Iran	Mean age = 14.69; Range: 11–19	Outcome
Sadeghi, Barahmand, and Roshannia, 2020 [72]	Iran	Mean age = 16.36; Range: 14–19	Outcome
Sagy and Adwan, 2006 [73]	Israel, Palestine	10th and 12th graders	Outcome
Sahin-Baltaci, 2018 [74]	Turkey	Mean age = 12.76; Range: 12–14	Outcome
Asia			
Bernardo, 2010 [75]	Philippines	Range: 16–23	Predictor
Botor, 2019 [76]	Philippines	Mean age = 17	Predictor
Chai, Kwok, and Gu, 2018 [77]	China–Hong Kong	Mean age = 10.06; Range: 8–13	Moderator
Chui and Wong, 2017 [78]	China–Hong Kong	Mean age = 14.2; Range: 10–19	Outcome
Izzaty and Ayriza, 2021 [79]	Indonesia	Mean age = 16.04; Range: 12–19	Outcome
Kwok and Gu, 2019 [80]	China–Hong Kong	Mean age = 13.86; Range: 10–18	Moderator
Ling, Huebner, Fu, Zeng, and He, 2016 [21]	China	Mean age = 15.85; Range: 13–18	Predictor
Ling, Huebner, Liu, Liu, Zhang, and Xiao, 2015 [81]	China	Mean age = 15.85; Range: 14–17	Predictor
Nazam and Husain, 2021 [82]	India	Mean age = 15.89; Range: 12–19	Validation of CHS
Nie, Teng, Bear, Guo, Liu, and Zhang, 2018 [83]	China	Mean age = 15.89; Range: 14–18	Mediator
Wong and Lim, 2009 [84]	Singapore	Mean age =15.6	Predictor
Xiang et al., 2022 [85]	China	Mean age = 16.45; Range: 11–24	Mediator
Yang et al., 2021 [86]	China	Mean age = 12.45; Range: 11–14	Validation of CHS
Yeung, Ho, and Mak, 2015 [87]	China–Hong Kong	Mean age = 15.19	Predictor

Note. * = Qualitative Method.

## Data Availability

No new data were created or analyzed in this study. Data sharing is not applicable to this article.

## References

[1] Scioli A., Ricci M., Nyugen T., Scioli E.R. (2011). Hope: Its nature and measurement. Psychol. Relig. Spiritual..

[2] Erikson E.H. (1953). Growth and crises of the healthy personality. II. Psyche.

[3] Snyder C.R., Harris C., Anderson J.R., Holleran S.A., Irving L.M., Sigmon S.T., Harney P. (1991). The will and the ways: Development and validation of an individual-differences measure of hope. J. Personal. Soc. Psychol..

[4] Snyder C.R., Rand K.L., Sigmon D.R., Snyder C.R., Lopez S., Snyder C., Lopez S.J. (2002). Hope theory: A member of the positive psychology family. Handbook of Positive Psychology.

[5] Overton W.F., Lerner R.M. (2012). Relational developmental systems: Paradigm for developmental science in the post-genomic era. Behav. Brain Sci..

[6] Callina K.S., Mueller M.K., Buckingham M.H., Gutierrez A.S., Bowers E.P., Geldhof G.J., Johnson S.K., Hilliard L.J., Hershberg R.M., Lerner J.V., Lerner R.M. (2015). Building hope for positive youth development: Research, practice, and policy. Promoting Positive Youth Development: Lessons from the 4-H Study.

[7] Schmid K.L., Lopez S.J. (2012). Positive pathways to adulthood: The role of hope in adolescents’ constructions of their futures. Adv. Child Dev. Behav..

[8] Brandtstädter J., Lerner R.M., Damon W. (2006). Action perspectives on human development. Theoretical Models of Human Development—Volume 1 of the Handbook of Child Psychology.

[9] Lerner J.V., Bowers E.P., Minor K., Boyd M.J., Mueller M.K., Schmid K.L., Napolitano C.M., Lewin-Bizan S., Lerner R.M., Lerner R.M., Easterbrooks M.A., Mistry J., Weiner I.B. (2013). Positive youth development: Processes, philosophies, and programs. Developmental Psychology. Volume 6 of the Handbook of Psychology.

[10] Lerner R.M. (2019). Frontiers in theory-predicated research in youth development: A commentary. J. Youth Dev..

[11] Lerner R.M., Bornstein M.H. (2021). Contributions of the specificity principle to theory, research, and application in the study of human development: A view of the issues. J. Appl. Dev. Psychol..

[12] Leman P.J., Smith E.P., Petersen A.C. (2017). Introduction to the Special Section of Child Development on Positive Youth Development in Diverse and Global Contexts. Child Dev..

[13] Lerner R.M., Lerner J.V., Bowers E., Geldhof G.J., Overton W.F., Molenaar P.C., Lerner R.M. (2015). Positive youth development and relational developmental systems. Theory and Method. Volume 1 of the Handbook of Child Psychology and Developmental Science.

[14] Geldhof G.J., Bowers E.P., Mueller M.K., Napolitano C.M., Callina K.S., Walsh K.J., Lerner J.V., Lerner R.M., Bowers E.P., Geldhof G.J., Johnson S.K., Hilliard L.J., Hershberg R.M., Lerner J.V., Lerner R.M. (2015). The Five Cs model of positive youth development. Promoting Positive Youth Development: Lessons from the 4-H Study.

[15] Edwards L.M., McClintock J.B., Gallagher M.W., Lopez S.J. (2018). A cultural context lens of hope. The Oxford Handbook of Hope.

[16] Nurmi J.E. (1991). How do adolescents see their future? A review of the development of future orientation and planning. Dev. Rev..

[17] Chang E.C., Banks K.H. (2007). The color and texture of hope: Some preliminary findings and implications for hope theory and counseling among diverse racial/ethnic groups. Cult. Divers. Ethn. Minor. Psychol..

[18] Dimitrova R., Wiium N. (2021). Handbook of Positive Youth Development: Research, Policy and Practice in a Global Context.

[19] Spencer M.B., Spencer T.R. (2014). Invited commentary: Exploring the promises, intricacies, and challenges to positive youth development. J. Youth Adolesc..

[20] Snyder C.R. (2002). Hope theory: Rainbows in the mind. Psychol. Inq..

[21] Ling Y., Huebner E.S., Fu P., Zeng Y., He Y. (2016). A person-oriented analysis of hope in Chinese adolescents. Personal. Individ. Differ..

[22] Snyder C.R. (2000). Handbook of Hope: Theory, Measures, and Applications.

[23] Lopez S.J., Synder C.R., Pedrotti J.T., Lopez S.J., Snyder C.R. (2003). Hope: Many definitions, many measures. Positive Psychological Assessment: A Handbook of Models and Measures.

[24] Brandtstädter J., Damon W., Lerner R.M. (1998). Action perspectives on human development. Theoretical Models of Human Development. Volume 1 of the Handbook of Child Psychology.

[25] Bukowski W.M., Laursen B., Rubin K.H. (2018). Handbook of Peer Interactions, Relationships, and Groups.

[26] Piaget J. (1970). The Science of Education and the Psychology of the Child.

[27] Smetana J.G., Robinson J., Rote W.M., Grusec J.E., Hastings P.D. (2015). Socialization in adolescence. Handbook of Socialization: Theory and Research.

[28] Schmid K.L., Phelps E., Kiely M.K., Napolitano C.M., Boyd M.J., Lerner R.M. (2011). The role of adolescents’ hopeful futures in predicting positive and negative developmental trajectories: Findings from the 4-H Study of Positive Youth Development. J. Posit. Psychol..

[29] Kenny M.E., Blustein D.L., Haase R.F., Jackson J., Perry J.C. (2006). Setting the stage: Career development and the student engagement process. J. Couns. Psychol..

[30] Gilman R., Dooley J., Florell D. (2006). Relative levels of hope and their relationship with academic and psychological indicators among adolescents. J. Soc. Clin. Psychol..

[31] Cedeno L.A., Elias M.J., Kelly S., Chu B.C. (2010). School violence, adjustment, and the influence of hope on low-income, African American youth. Am. J. Orthopsychiatry.

[32] Valle M.F., Huebner E.S., Suldo S.M. (2006). An analysis of hope as a psychological strength. J. Sch. Psychol..

[33] Hollingsworth D.W., Wingate L.R., Tucker R.P., O’Keefe V.M., Cole A.B. (2016). Hope as a moderator of the relationship between interpersonal predictors of suicide and suicidal thinking in African Americans. J. Black Psychol..

[34] Bornstein M.H. (2017). The specificity principle in acculturation science. Perspect. Psychol. Sci..

[35] Lerner J.V., Bowers E.P., Minor K., Boyd M.J., Mueller M.K., Schmid K.L., Lerner R.M., Lerner R.M., Easterbrooks M.A., Mistry J., Weiner I.B. (2012). Positive youth development: Processes, philosophies, and programs. Developmental Psychology. Volume 6 of the Handbook of Psychology.

[36] Lerner R.M., Lerner J.V., Murry V.M., Smith E.P., Bowers E.P., Geldhof G.J., Buckingham M.H. (2021). Positive youth development in 2020: Theory, research, programs, and the promotion of social justice. J. Res. Adolesc..

[37] Lo Iaconoa L., Carola V. (2018). The impact of adolescent stress experiences on neurobiological development. Semin. Cell Dev. Biol..

[38] Jovanović V. (2013). Evaluation of the Children’s Hope Scale in Serbian adolescents: Dimensionality, measurement invariance across gender, convergent and incremental validity. Child Indic. Res..

[39] Adelabu D.H. (2008). Future time perspective, hope, and ethnic identity among African American adolescents. Urban Educ..

[40] Davis-Maye D., Perry T.E. (2007). Momma’s girl: The significance of maternal figure support in the development of hope for African American girls. J. Hum. Behav. Soc. Environ..

[41] Dixson D.D., Keltner D., Worrell F.C., Mello Z. (2018). The magic of hope: Hope mediates the relationship between socioeconomic status and academic achievement. J. Educ. Res..

[42] Edwards L.M., Ong A.D., Lopez S.J. (2007). Hope measurement in Mexican American youth. Hisp. J. Behav. Sci..

[43] Harley D. (2015). Perceptions of hopelessness among low-income African-American adolescents through the lens of photovoice. J. Ethn. Cult. Divers. Soc. Work..

[44] McCoy H., Bowen E.A. (2015). Hope in the social environment: Factors affecting future aspirations and school self-efficacy for youth in urban environments. Child Adolesc. Soc. Work. J..

[45] Roesch S.C., Duangado K.M., Vaughn A.A., Aldridge A.A., Villodas F. (2010). Dispositional hope and the propensity to cope: A daily diary assessment of minority adolescents. Cult. Divers. Ethn. Minor. Psychol..

[46] Shadlow J.O., Boles R.E., Roberts M.C., Winston L. (2015). Native American children and their reports of hope: Construct validation of the children’s hope scale. J. Child Fam. Stud..

[47] Twyford J.M., Dowdy E., Sharkey J.D. (2014). Implications of self-reported levels of hope in Latino and Latina youth on probation. J. Juv. Justice.

[48] Vela J.C., Lenz A.S., Sparrow G.S., Gonzalez S.L. (2017). Using a positive psychology and family framework to understand Mexican American adolescents’ college-going beliefs. Hisp. J. Behav. Sci..

[49] Alfieri S., Quartiroli A., Baumann D. (2021). Adaptation of the Snyder’s dispositional Hope Scale for Italian adolescents. Curr. Psychol..

[50] Falanga R., De Caroli M.E., Sagone E., Indiana M.L. (2020). Are humor styles predictors of hope? Sex and age differences in Italian adolescents and young adults. Int. J. Psychol. Psychol. Ther..

[51] Marques S.C., Lopez S.J., Pais-Ribeiro J.L. (2011). “Building hope for the future”: A program to foster strengths in middle-school students. J. Happiness Stud..

[52] Marques S.C., Lopez S.J., Mitchell J. (2013). The role of hope, spirituality and religious practice in adolescents’ life satisfaction: Longitudinal findings. J. Happiness Stud..

[53] Marques S.C., Pais-Ribeiro J., Lopez S.J., Brdar I. (2011). Further evaluation of the test-retest reliability of the Children Hope Scale and Students’ Life Satisfaction Scale. The Human Pursuit of Well-Being.

[54] Martins A.R., Crespo C., Salvador Á., Santos S., Carona C., Canavarro M.C. (2018). Does hope matter? Associations among self-reported hope, anxiety, and health-related quality of life in children and adolescents with cancer. J. Clin. Psychol. Med. Settings.

[55] Merkaš M., Brajša-Žganec A. (2011). Children with different levels of hope: Are there differences in their self-esteem, life satisfaction, social support, and family cohesion?. Child Indic. Res..

[56] Santilli S., Marcionetti J., Rochat S., Rossier J., Nota L. (2017). Career adaptability, hope, optimism, and life satisfaction in Italian and Swiss adolescents. J. Career Dev..

[57] Guse T., Vermaak Y. (2011). Hope, psychosocial well-being and socioeconomic status among a group of South African adolescents. J. Psychol. Afr..

[58] Guse T., de Bruin G.P., Kok M. (2016). Validation of the Children’s Hope Scale in a Sample of South African Adolescents. Child Indic. Res..

[59] Isaacs S.A., Savahl S. (2014). A qualitative inquiry investigating adolescents’ sense of hope within a context of violence in a disadvantaged community in Cape Town. J. Youth Stud..

[60] Nalkur P.G. (2009). Adolescent hopefulness in Tanzania: Street youth, former street youth, and school youth. J. Adolesc. Res..

[61] Savahl S., Isaacs S., Adams S., Carels C.Z., September R. (2013). An exploration into the impact of exposure to community violence and hope on children’s perceptions of well-being: A South African perspective. Child Indic. Res..

[62] Wilson A., Somhlaba N. (2016). Psychological well-being in the context of adversity: Ghanaian adolescents’ experiences of hope and life satisfaction. Afr. Today.

[63] Ciarrochi J., Heaven P.C., Davies F. (2007). The impact of hope, self-esteem, and attributional style on adolescents’ school grades and emotional well-being: A longitudinal study. J. Res. Personal..

[64] Ciarrochi J., Parker P., Kashdan T.B., Heaven P.C., Barkus E. (2015). Hope and emotional well-being: A six-year study to distinguish antecedents, correlates, and consequences. J. Posit. Psychol..

[65] Heaven P., Ciarrochi J. (2008). Parental styles, gender and the development of hope and self-esteem. Eur. J. Personal..

[66] Phan H.P. (2013). Examination of self-efficacy and hope: A developmental approach using latent growth modeling. J. Educ. Res..

[67] Braun-Lewensohn O., Sagy S. (2010). Sense of coherence, hope and values among adolescents under missile attacks: A longitudinal study. Int. J. Child. Spiritual..

[68] Çelik D.A., Çetin F., Tutkun E. (2015). The role of proximal and distal resilience factors and locus of control in understanding hope, self-esteem and academic achievement among Turkish pre-adolescents. Curr. Psychol..

[69] Karababa A. (2020). The moderating role of hope in the relationship between maladaptive perfectionism and anxiety among early adolescents. J. Genet. Psychol..

[70] Kemer G., Atik G. (2012). Hope and social support in high school students from urban and rural areas of Ankara, Turkey. J. Happiness Stud..

[71] Khodarahimi S. (2014). The role of family violence on mental health and hopefulness in an Iranian adolescent sample. J. Fam. Violence.

[72] Sadeghi M., Barahmand U., Roshannia S. (2020). Differentiation of self and hope mediated by resilience: Gender differences. Can. J. Fam. Youth J. Can. Fam. Jeun..

[73] Sagy S., Adwan S. (2006). Hope in times of threat: The case of Israeli and Palestinian youth. Am. J. Orthopsychiatry.

[74] Sahin-Baltacı H. (2018). Comparison of hope and life satisfaction levels of Turkish and American middle school students. Eurasian J. Educ. Res..

[75] Bernardo A.B. (2010). Extending hope theory: Internal and external locus of trait hope. Personal. Individ. Differ..

[76] Botor N.J. (2019). Hope predicts happiness with life, personal well-being, and resilience among selected school-going Filipino adolescents. Int. J. Sci. Basic Appl. Res..

[77] Chai W.Y., Kwok S.Y., Gu M. (2018). Autonomy-granting parenting and child depression: The moderating roles of hope and life satisfaction. J. Child Fam. Stud..

[78] Chui W.H., Wong M.Y. (2017). Avoiding disappointment or fulfilling expectation: A study of gender, academic achievement, and family functioning among Hong Kong adolescents. J. Child Fam. Stud..

[79] Izzaty R.E., Ayriza Y. (2021). Parental bonding as a predictor of hope in adolescents. Psikohumaniora J. Penelit. Psikol..

[80] Kwok S.Y., Gu M. (2019). Childhood neglect and adolescent suicidal ideation: A moderated mediation model of hope and depression. Prev. Sci..

[81] Ling Y., Huebner E.S., Liu J., Liu W.L., Zhang J., Xiao J. (2015). The origins of hope in adolescence: A test of a social–cognitive model. Personal. Individ. Differ..

[82] Nazam F., Husain A. (2021). Psychometric revalidation of Children’s Hope Scale among Indian adolescents. Asia Pac. Soc. Sci. Rev..

[83] Nie Q., Teng Z., Bear G.G., Guo C., Liu Y., Zhang D. (2019). Hope as mediator between teacher–student relationships and life satisfaction among Chinese adolescents: A between-and within-person effects analysis. J. Happiness Stud..

[84] Wong S., Lim T. (2009). Hope versus optimism in Singaporean adolescents: Contributions to depression and life-satisfaction. Personal. Individ. Differ..

[85] Xiang G., Li Q., Du X., Liu X., Xiao M., Chen H. (2022). Links between family cohesion and subjective well-being in adolescents and early adults: The mediating role of self-concept clarity and hope. Curr. Psychol..

[86] Yang Q., Ling Y., Huebner E.S., Zeng Y., Liu C. (2021). Assessing the measurement invariance of the Children’s Hope Scale in Chinese and American adolescents. J. Personal. Assess..

[87] Yeung D.Y., Ho S.M., Mak C.W. (2015). Brief report: Attention to positive information mediates the relationship between hope and psychosocial well-being of adolescents. J. Adolesc..

[88] Special Eurobarometer (2005). European Commission Eurobarometer: Social values, Science and Technology. http://ec.europa.eu/public_opinion/archives/ebs/ebs_225_report_en.pdf.

[89] Haj-Yahia M.M. (2001). The incidence of witnessing interparental violence and some of its psychological consequences among Arab adolescents. Child Abus. Negl..

[90] Haj-Yahia M.M., Musleh K., Haj-Yahia Y.M. (2002). The incidence of adolescent maltreatment in Arab society and some of its psychological effects. J. Fam. Issues.

[91] Snyder C.R., Cheavens J., Sympson S.C. (1997). Hope: An individual motive for social commerce. Group Dyn. Theory Res. Pract..

[92] Lerner R.M., Bowers E.P., Geldhof G.J., Gestsdóttir S., DeSouza L. (2012). Promoting positive youth development in the face of contextual changes and challenges: The roles of individual strengths and ecological assets. New Dir. Youth Dev..

[93] Bowers E.P., Winburn E.N., Sandoval A.M., Clanton T. (2020). Culturally relevant strengths and positive development in high achieving youth of color. J. Appl. Dev. Psychol..

[94] Simpkins S.D., Riggs N.R., Ngo B., Vest Ettekal A., Okamoto D. (2017). Designing culturally responsive organized after-school activities. J. Adolesc. Res..

[95] Zaff J.F. (2011). A cease-and-desist order for school reform: It is time for educational transformation. Appl. Dev. Sci..

[96] Kubisch A., Auspos P., Brown P., Dewar T. (2010). Voices from the Field III: Lessons and Challenges from Two Decades of Community Change Efforts.

